# Skin Resident Memory T Cells May Play Critical Role in Delayed-Type Drug Hypersensitivity Reactions

**DOI:** 10.3389/fimmu.2021.654190

**Published:** 2021-08-23

**Authors:** Elisa Maria Schunkert, Pranali Nitin Shah, Sherrie Jill Divito

**Affiliations:** Department of Dermatology, Brigham & Women’s Hospital, Harvard Medical School, Boston, MA, United States

**Keywords:** adverse drug reactions, delayed-type drug hypersensitivity reactions, fixed drug eruption, maculopapular exanthem, drug reaction with eosinophil and systemic symptoms, Stevens-Johnson syndrome, tissue-resident memory T cells, toxic epidermal necrolysis

## Abstract

Delayed-type drug hypersensitivity reactions (dtDHR) are immune-mediated reactions with skin and visceral manifestations ranging from mild to severe. Clinical care is negatively impacted by a limited understanding of disease pathogenesis. Though T cells are believed to orchestrate disease, the type of T cell and the location and mechanism of T cell activation remain unknown. Resident memory T cells (T_RM_) are a unique T cell population potentially well situated to act as key mediators in disease pathogenesis, but significant obstacles to defining, identifying, and testing T_RM_ in dtDHR preclude definitive conclusions at this time. Deeper mechanistic interrogation to address these unanswered questions is necessary, as involvement of T_RM_ in disease has significant implications for prediction, diagnosis, and treatment of disease.

## Introduction

Delayed-type drug hypersensitivity reactions (dtDHR) are a significant public health problem with potential for high morbidity and mortality and considerable cost to healthcare systems (1–5). Skin is the most commonly affected organ with severity ranging from a mild exanthem (maculopapular drug eruption, MPE), to life-threatening blistering and sloughing of skin (Stevens-Johnson syndrome/toxic epidermal necrolysis, SJS/TEN), with potential for significant visceral involvement (drug reaction with eosinophilia and systemic symptoms, DRESS). The pathobiology of dtDHR remains poorly understood, impeding prediction and prevention, diagnosis, identification of culprit drug, and effective treatment. DtDHR are immune-mediated adverse drug reactions that typically appear days to weeks after drug exposure (3). The timing of onset combined with epidemiological, histopathological and laboratory data incriminate T cells as primary drivers of disease, however, the type of pathogenic T cell and the location and mechanism of its activation are unknown. Tissue-resident memory T cells (T_RM_) are an increasingly appreciated unique population of T cells that persist long-term in peripheral tissues including skin (6, 7). Critical roles for skin T_RM_ in cutaneous health and several inflammatory skin diseases have been identified (6, 8), raising the question of whether skin T_RM_ contribute to or cause dtDHR. This mini-review aims to provide the reader with a clearer understanding of these enigmatic cells, evidence supporting skin T_RM_ involvement in dtDHR pathogenesis, and current barriers limiting investigation in this field.

## An Overview of Skin T_RM_ Biology

### T Cell Classification and Identification

T cell classification is increasingly complex due to significant heterogeneity within T cell compartments and lack of consensus on nomenclature. Differences between mouse and human contribute further to confusion. This review therefore focuses on the most salient features required to understand skin T_RM_ in the context of dtDHR.

Mature T cells are broadly classified into naïve, effector, and memory subsets. When a naïve T cell is stimulated with cognate antigen, changes in surface expression of multiple proteins occur that are easily assayed by flow cytometry. In both humans and mice, cell surface expression of CD44 is increased, thereby differentiating naïve T cells (CD44^low^) from effector and memory T cells (CD44^high^) (9, 10). Additionally, activated T cells decrease surface expression of CD62L (L-selectin) and the chemokine receptor CCR7, and transiently up-regulate CD69 (11–14). In addition to these phenotypic changes, T cell proliferation, migration to the site of inflammation, and acquisition of effector function occur, ultimately resulting in resolution of the antigenic insult (14, 15). Once resolved, the effector phase of the response concludes with T cell contraction. However, a small population of antigen-specific memory T cells survive that are capable of responding faster and more robustly upon re-exposure to their cognate antigen (14–16). In humans, different isoforms of CD45 expressed on T cells are generally helpful in distinguishing naïve T cells, CD45RA^+^, from memory T cells, which are typically CD45RO^+^ (17).

Memory T cells were previously classified into two major subsets: central (T_CM_) and effector (T_EM_) memory T cells (18). T_CM_ reside predominantly in secondary lymphoid organs due to their surface expression of CCR7 and CD62L and carry a high proliferative capacity upon antigen re-exposure, but lack the ability to rapidly produce effector molecules (18). Comparatively, T_EM_ lack CCR7 and have low CD62L expression allowing them to circulate throughout the body during steady-state (18). In keeping with their role as circulating sentinels, they are capable of rapid effector function (18). More recently, T_RM_ have been appreciated as a unique memory T cell population playing key roles in health and disease. T_RM_ persist in robust numbers long-term in peripheral tissues despite absence of inflammation. Barrier sites including skin, gut, liver, lung, and mucosa are the main tissues containing large numbers of T_RM_ (19–24), presumably due to the constant barrage of these tissues with environmental antigens. Phenotypically, T_RM_ have low/absent expression of CD62L and CCR7 (16, 22), which helps prevent migration to secondary lymphoid organs (25, 26), and low expression of the transcription factor KLF2 and the protein S1P1 while maintaining surface expression of CD69, which further facilitates retention in peripheral tissue (27–30).

Skin T_RM_ consist of both CD4^+^ and CD8^+^ type T cells, each with variable phenotype and distribution throughout skin. A subset of epidermal CD4^+^ and CD8^+^ T_RM_ express CD103, the α_E_ subunit of α_E_β_7_ integrin, which helps anchor T_RM_ to epithelial cells expressing E-cadherin (29–32). The majority of T_RM_ in healthy human skin reside in the dermis and are phenotypically CD4^+^CD103^-^ (29). Skin T_RM_ commonly express on their surface the skin homing molecule cutaneous lymphocyte antigen (CLA) (22) and variably the chemokine receptors CCR4, CCR6, CCR8, CCR10, and CXCR6 (22, 33–37). Taken together, T_RM_ are overall best phenotypically identified in healthy human skin as CD3^+^, CD4^+^ or CD8^+^, CD45RO^+^CD69^+^CLA^+^CCR7^-^CD62L^low^ and either CD103^+^ or CD103^–^.

### T_RM_ Maintain Skin Health

T_RM_ play an important role in immunity. Protective skin T_RM_ can be generated in mice by immunizing with vaccinia virus (38, 39) or HSV (40, 41), and these T_RM_ clear pathogens faster and more effectively than, or even in the absence of, circulating T cells (38–41). Skin T_RM_ in humans likewise appear to effectively prevent cutaneous infection in the absence of circulating T cells, as patients depleted of circulating T cells by alemtuzumab, an anti-CD52 antibody, do not experience increased rates of infection (42). T_RM_ provide surveillance by actively patrolling skin (43, 44) and are capable of rapid and potent pro-inflammatory cytokine release (29), though data suggest that rapid cytotoxicity may be constrained by PD-1 signaling (45). Surface expression of the integrin CD49a reportedly denotes functionality of skin T_RM_, with CD8^+^CD49a^+^ T_RM_ capable of IFNγ production and cytotoxicity, while CD8^+^CD49a^-^ T_RM_ are polarized toward IL-17 production (46, 47). Notably, localized skin infection generates T_RM_ not only at the site of infection but potentially at distant skin sites as well, particularly in the setting of repeat inoculation (39, 48, 49). Repeated exposure to a diversity of microbes could therefore generate an army of sentinel antigen-specific T cells across the expanse of skin, poised to rapidly defend against infection.

### Skin T_RM_ Are Instrumental in Several Inflammatory Skin Diseases

Increasing data support skin T_RM_ as potentially causal or contributory to acute and chronic inflammatory skin conditions. In atopic dermatitis and psoriasis, T Cell Receptor (TCR) sequencing identified that pathogenic T cell clones persisted in skin at the site of resolved lesions supporting their classification as skin T_RM_ (50, 51). T cells remaining in resolved psoriatic lesional skin retained the propensity to produce psoriasis-inducing cytokines, explaining predisposition to disease recurrence at the same skin location after treatment discontinuation (51, 52), and pathogenic T_RM_ residing in non-lesional skin from psoriasis patients are capable of eliciting a psoriasiform reaction when stimulated ex vivo (53).

Moreover, human studies suggest a role for skin T_RM_ in acute graft-versus-host disease (GVHD), as skin T cells were shown to survive robust chemotherapy ± total body irradiation and were present and activated during acute skin GVHD (54). Findings were complemented by a humanized mouse model showing that human skin T_RM_ could mediate a GVHD-like dermatitis in the absence of donor T cells (54). These findings have direct implications for dtDHR, as acute skin GVHD clinically and histologically mirrors MPE in mild form and SJS/TEN in severe form.

Finally, skin T_RM_ have been directly implicated in allergic contact dermatitis (ACD), another form of cutaneous delayed-type hypersensitivity reaction. In an ACD mouse model, increased allergen dose and number of exposures resulted in increased frequency of epidermal CD8^+^ T_RM_ and worsened disease (55), and skin T_RM_ were observed to mediate allergic reactions upon allergen re-exposure in the absence of circulating T cells (49, 56). Allergen exposure induced antigen-specific T_RM_ locally and at distant skin sites, and concurrently antigen-specific T_CM_ in draining and distant lymph node, all bearing identical TCR, indicating that the newly generated memory T cells derived from a common clone (49). Interestingly, disease was exacerbated by checkpoint inhibitor antagonists which stimulated T_RM_ effector function, intimating that checkpoint inhibitors do constrain the T_RM_ response (56).

## Do T_RM_ Mediate dtDHR?

### T Cells in dtDHR

Ample evidence implicates T cell mediation of dtDHR. T cell infiltrate and pro-inflammatory molecules commonly attributed to effector T cells are observed within biopsies or blister fluid from affected skin (57, 58). Additionally, drug-specific T cells from peripheral blood have been identified by clonality studies, and in some diseased patients T cells from peripheral blood respond to drug in lymphocyte stimulation tests (59, 60). Moreover, several HLA associations have been identified that predispose patients to specific drug reactions (61–63). Prominent examples include HLA*B57:01 which predisposes patients taking the drug abacavir to DRESS (64), and HLA*B15:02 which predisposes patients to SJS/TEN if administered carbamazepine (65). T cell effector functions in dtDHR are increasingly appreciated, particularly in SJS/TEN, as CD8^+^ T cell produced cytotoxic granules and soluble granulysin are thought to mediate keratinocyte death (58, 66). Comparatively, MPE is largely considered CD4^+^ T cell/cytokine driven. Contradictory findings have been observed in DRESS, with some research supporting Th2 polarization, while other studies have observed cytotoxic CD8^+^ T cells and IFNγ and TNFα signatures (66). Despite significant advancements in the above areas of research, the type of T cell mediating disease (effector, central *vs* effector *vs* resident memory) and the location and mechanism by which T cells are stimulated against drug remains largely unknown.

### Evidence Supporting a Role for T_RM_ in dtDHR

Studies directly investigating T_RM_ in dtDHR are sparse, though there is increasing circumstantial evidence suggesting that skin T_RM_ may be critical players in disease. Anecdotally, patients with few circulating lymphocytes secondary to chemotherapy are capable of developing dtDHRs. Iriki et al. reported a case of SJS/TEN in a patient with severe lymphopenia secondary to chemotherapy. They demonstrated by immunofluorescence microscopy of affected skin the presence of CD45RO^+^CD69^+^ CD4^+^ and CD8^+^ T cells despite the lack of circulating T cells (67). The presumption is that disease resulted from skin T_RM_ that survived chemotherapy, while naïve and central and effector memory T cells were depleted by the treatment. However, the possibility that a low number of T cells survived chemotherapy in secondary lymphoid organs and were recruited to skin to mediate disease has not been ruled out.

A recent publication by Trubiano et al. investigated skin T_RM_ in patients with MPE and DRESS (68). The study reported increased number of CD45RO^+^CD69^+^ CD4^+^ and CD8^+^ T cells in skin after resolution of DRESS in one patient. In addition, the authors performed a localized drug challenge by intradermally injecting culprit drug into skin of patients with history of MPE or DRESS. Skin biopsies performed acutely after challenge during active localized inflammation and again 8 weeks after resolution demonstrated accumulation of CD45RO^+^CD69^+^CD103^+^ CD4^+^ and CD8^+^ T cells at the 8-week time point. The increased frequency of cells with this phenotype supports that skin T_RM_ are generated by systemic disease and localized drug exposure (68). Similarly, patch testing suspected culprit drug in some cases induces skin inflammation in patients with history of dtDHR, affirming that drug-specific memory T cells are present in skin after disease resolution. For example, patch testing against abacavir has high sensitivity in patients with history of DRESS who express HLA-B*57:01 (69). Comparatively, drug naïve HLA-B*57:01 positive patients were demonstrated to have circulating abacavir-reactive T cells yet patch test negative, reinforcing the idea that drug exposure induces skin T_RM_ (70). Of note though, patch testing reportedly has low sensitivity for dtDHR reactions (71–73), raising the question of whether skin T_RM_ accumulation is dependent on type of dtDHR, culprit drug, and/or presence of specific HLA alleles, or alternatively low sensitivity may be secondary to a technical issue for example suboptimal drug concentration or vehicle applied during testing.

Lastly and perhaps most intriguingly, the idea that T_RM_-mediated immunity is restrained by immune checkpoint signaling further bolsters the link between skin T_RM_ and dtDHR (56). The coinhibitory molecules CTLA-4 and PD-1 have been linked to hypersensitivity reactions from multiple drugs in both human and animal studies (74). Clinically, immune checkpoint inhibitors (ICI) can induce dtDHR-like reactions, most notably an SJS/TEN-like phenomenon (75, 76). It is currently unknown whether these reactions develop due to ICI-provoked loss of tolerance to a concurrently administered drug or bystander activation in the setting of a robust anti-tumor T cell response. Further investigation of these reactions has substantial potential to illuminate pathobiology of dtDHR.

### The Curious Case of Fixed Drug Eruptions

Perhaps the best case for skin T_RM_ mediating at least one form of dtDHR is that of fixed drug eruption (FDE). Remarkably, FDE lesions reappear at the exact same skin site upon re-exposure to causative drug. Studies in resolved FDE lesions in human skin showed that intraepidermal CD8^+^ T cells constitutively expressed CD69 in the absence of inflammation, and upon drug challenge produced IFNγ (77). Though these findings are consistent with skin T_RM_, these T cells expressed primarily CD45RA^+^ rather than CD45RO^+^ (77). A separate unique subset of memory T cells termed T_emra_ can express CD45RA rather than CD45RO (18, 78, 79), but in this study, the intraepidermal CD8^+^ T cells lacked CD57, a marker suggestive of T_emra_. This peculiar finding is yet to be resolved but highlights the phenotypic heterogeneity of memory T cell populations and a major challenge to accurately identifying T_RM_.

## Insight Into Potential Mechanisms of Drug-Reactive T Cell Stimulation

**Figure 1:**
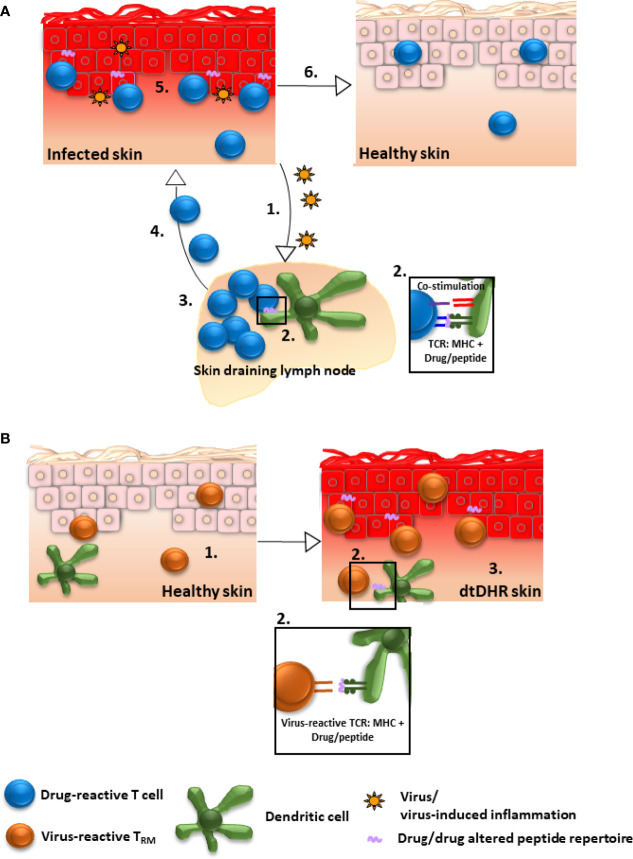
Possible mechanisms by which drug-reactive T_RM_ could be generated in skin. **(A)** Drug/drug altered peptide repertoire is presented to drug-reactive T cells and concurrent viral infection/reactivation provides sufficient co-stimulation to break T cell tolerance to drug: 1. Inflammatory mediators secondary to infection stimulate dendritic cells. 2. Activated dendritic cells in skin draining lymph nodes present drug/drug altered peptide repertoire to naïve drug-reactive T cells and provide ample co-stimulation resulting in T cell priming. 3. Primed T cells proliferate and differentiate into effector cells, 4. migrate to skin, and 5. mediate damage as a primary immune response. 6. Despite resolution of inflammation, drug-reactive T_RM_ remain in skin, poised to mediate repeat dtDHR upon re-exposure to drug. Alternatively, factors other than viral infection, for example altered drug metabolism or reduced clearance, could potentially lead to T cell activation (not shown). **(B)** Drug-reactive T cells are cross-reactive to viral epitopes: 1. Virus-specific T_RM_ accumulate in skin as a consequence of prior infection. 2. These virus-reactive T cells are capable of recognizing (cross-reacting to) drug/drug altered peptide repertoire presented by MHC on the surface of skin dendritic cells, macrophages and/or keratinocytes resulting in T_RM_ stimulation. 3. The stimulated T_RM_ produce pro-inflammatory molecules inducing DRESS, or potentially other dtDHR.

The fact that only a subset of patients expressing a predisposing HLA allele develop dtDHR upon exposure to the associated drug (80) suggests that a second “X” factor is necessary for disease. Evidence supports that viral infection or reactivation, particularly with herpes family viruses (HHV6, HHV7, EBV, CMV), may be this “X” factor (80–83). Presumably, the viral infection provides co-stimulation necessary to break T cell tolerance to drug (**Figure 1A**). In this scenario, drug-reactive naïve T cells could be primed in skin draining lymph nodes by dendritic cells activated by virally induced inflammation, the drug-reactive effector T cells migrate to skin and mediate disease as a primary immune response, then after contraction, a population of drug-reactive T_RM_ remain in skin, capable of mediating a reaction upon drug re-exposure. Though most research has focused on infection/reactivation of herpes family viruses in the context of DRESS, similar observations have been made in MPE, the classic example being ampicillin-induced MPE in the context of EBV mononucleosis (84), and in two publications in SJS/TEN (one reporting HHV6 reactivation and one EBV reactivation) (85, 86). Another potential “X” factor could be increased drug levels secondary to altered drug metabolism or reduced drug clearance. For example, data demonstrate that reduced renal function predisposes to allopurinol-induced severe dtDHR (87, 88).

Alternatively, virus-specific skin T_RM_ generated in response to a previous viral infection could potentially *cross*-*react* to drug/drug altered peptide repertoire presented by self-MHC expressed on skin antigen presenting cells or keratinocytes (80) (**Figure 1B**). In support of this, activated CD8^+^ T cells mediating cutaneous and visceral symptoms of DRESS were shown to be EBV reactive (81), and HIV-specific memory T cell clones were demonstrated to react *in vitro* against abacavir-altered peptide repertoire presented in the context of HLA-B*57:01 (89) Whether cross-reactivity is causal to dtDHR other than DRESS remains to be elucidated.

Either scenario may help explain the link between skin T_RM_ and dtDHR with systemic manifestations. Research supports that cutaneous viral infection and ACD can generate clonal T_RM_ and T_CM_ simultaneously in local and distant skin and secondary lymphoid organs (39, 48, 49) and potentially antigen-specific T_RM_ in other tissues as well (90). In support of this concept, identical pathogenic clones were identified in blood, skin, liver, and lung of one patient with DRESS and blood, skin, and liver in a second patient (81).

Moreover, either above scenario could account for development of dtDHR upon first exposure to a drug. Lucas et al. demonstrated that drug naïve, HLA-B*57:01 positive patients contained both naïve and memory T cells in circulation capable of responding to abacavir upon *in vitro* stimulation (91). Presumably, rapid development of DRESS upon first drug exposure reflects mediation by cross-reactive memory T cells, while slower onset points toward priming of a primary drug-specific immune response. Whether this phenomenon occurs in other types of dtDHR remains unknown.

## Limitations to the Study of Skin T_RM_ in dtDHR

Several barriers impede the study of skin T_RM_ in dtDHR. First, many studies rely on limited phenotypic analysis by flow cytometry or tissue immunostaining to identify skin T_RM_. Flow cytometry commonly requires digestion and disaggregation of skin by chemical and/or physical measures resulting in loss of spatial information and potential alteration of surface marker expression (92–94). Standard tissue immunostaining overcomes these barriers but is limited to co-staining of maximum 3 antigens, and some antigens are difficult to reliably stain in formalin-fixed tissue. Further, because no single marker or combination of markers defines all skin T_RM_, cursory phenotypic analysis can be misleading. This is especially true during active inflammation, as CD69 can be expressed by other activated T cell populations, and as alluded to above, CD45RO^+^CD45RA^-^ phenotype is not universal for all memory T cells (18, 78, 79, 95). More perplexing, the definition that T_RM_ remain resident in peripheral tissue without recirculating has been called into question. Klicznik et al. reported that CD4^+^CD69^+^CD103^+^ T_RM_ are capable of downregulating the tissue-retention marker CD69 and entering circulation (96). Moreover, elegant work by Buggert et al., transforms the current construct of memory T cell classification with observations that well-differentiated cytolytic memory CD8^+^ T cells remain in intravascular circulation under steady-state conditions, while less differentiated non-cytolytic non-resident memory CD8^+^ T cells recirculate between lymphoid and non-lymphoid tissues such as liver and gut (97). Whether these findings apply to skin T_RM_ in steady-state or during dtDHR is unknown, though one can imagine that preferential maintenance of less differentiated non-cytolytic T cells rather than cytolytic T cells in tissue could be a potential means of preventing bystander tissue damage.

A second major barrier is that researchers have limited ability to investigate the immunologic events preceding clinical manifestations of dtDHR due to either lack of predictability or that it may be unsafe to (re)expose a patient to drug in the event that a reaction is predictable. Prospective studies of active disease can be equally challenging particularly for rarer forms of dtDHR and for severe forms that may require rapid initiation of treatment prior to sampling. This type of clinical scenario is where mouse models become essential. Historically, dtDHR research has been hampered by the lack of mouse models that meaningfully recapitulate disease, but recent advances have been achieved by administering drug to mice transgenically expressing predisposing HLA alleles (98, 99). Though generation of, or mediation by, skin T_RM_ has not yet been tested in these mice, these models provide for the first time a platform allowing for deeper mechanistic interrogation of T_RM_ in disease.

## Conclusions

A contribution from skin T_RM_ in dtDHR pathogenesis is an intriguing possibility with potential to shed light on a number of unanswered questions in the field. From a basic immunology perspective, it would address not only which T cells mediate disease but would also provide insight into mechanism(s) of T cell activation against drug. From a clinical perspective, it could aid predictability and diagnosis, allow for development of novel strategies to identify culprit drug, and provide insight into alternative approaches to treatment. Despite current obstacles to the study of T_RM_ in dtDHR, there is an increasingly strong framework in place and clearly a clinical need to justify further investigation.

## Author Contributions

ES prepared the manuscript with assistance from PS. SD supervised the project and oversaw the authoring and revision of the manuscript. All authors contributed to the article and approved the submitted version.

## Funding

This work was funded by the National Institutes of Health DP5 OD023091 and R21 AI150657 (SD) the German Research Foundation, DFG Project number: 423175926, GZ: SCHU 3377/1-1 (ES) and the Sun Pharma/SID Innovation Research Fellowship (PS).

## Conflict of Interest

The authors declare that the research was conducted in the absence of any commercial or financial relationships that could be construed as a potential conflict of interest.

## Publisher’s Note

All claims expressed in this article are solely those of the authors and do not necessarily represent those of their affiliated organizations, or those of the publisher, the editors and the reviewers. Any product that may be evaluated in this article, or claim that may be made by its manufacturer, is not guaranteed or endorsed by the publisher.
